# Genome-Wide Identification and Expression Analysis of the *GDPD* Gene Family in Cucumber (*Cucumis sativus* L.)

**DOI:** 10.3390/cimb48060602

**Published:** 2026-06-05

**Authors:** Shanyu Li, Xinjie Zhang, Leiming Cao, Yang Zhou, Ruitong Zhang, Lisi Jiang, Wei Fu

**Affiliations:** College of Life Science, Shenyang Normal University, Shenyang 110034, China; 15524253653@163.com (S.L.); 15041158087@163.com (X.Z.); 17263360512@163.com (L.C.); zy1925816377@163.com (Y.Z.); zhangru1t@163.com (R.Z.)

**Keywords:** *GDPD* gene family, cucumber, abiotic stress

## Abstract

Glycerophosphate diester phosphodiesterase (GDPD) catalyzes the decomposition of glycerophosphate diester into sn-glycerol-3-phosphate and corresponding alcohols. In this study, six GDPD genes were identified in the cucumber genome, named *CsGDPD1* to *CsGDPD6*, and distributed on chromosomes 1, 3, 4, 5, 6, and 7. All six proteins exhibited similar predicted three dimensional structures, suggesting conserved biochemical functions. Phylogenetic and dN/dS selection pressure analyses revealed that *CsGDPD* genes are evolutionarily close to their *Arabidopsis* homologs and have evolved under purifying selection, indicating functional conservation. Synteny analysis identified five collinear gene pairs between cucumber and *Arabidopsis*, but no synteny with rice. Promoter cis-acting element analysis showed the presence of multiple stress- and hormone-responsive elements. Tissue-specific expression profiling demonstrated that *CsGDPD1*, *CsGDPD2*, and *CsGDPD6* are broadly expressed across tissues, whereas *CsGDPD4* and *CsGDPD5* show preferential expression in reproductive organs. qRT-PCR under drought and salt stress, with or without the plant growth promoting rhizobacterium GD17, revealed that drought alone upregulates all *CsGDPD* genes; PGPR-GD17 alone (+PGPR) suppresses their expression; and combined PGPR + Drought leads to synergistic suppression. Under salt stress, *CsGDPD5* was dramatically upregulated (20-fold), and PGPR-GD17 partially reversed salt induced changes. These results provide a comprehensive foundation for understanding the evolutionary and functional roles of the GDPD gene family in cucumber stress responses.

## 1. Introduction

Lipids are major membrane components. Recent studies on lipid signaling and metabolism have revealed their essential roles in plant stress tolerance, including drought, heat, cold, salinity, and pathogen attack [1]. Lipid remodeling is also crucial for alleviating nutrient deprivation, particularly phosphate (Pi) deficiency. Phosphorus (P) is an essential macronutrient for plant growth, participating in multiple critical physiological processes such as cell structure construction, energy generation, metabolic regulation, and signal transduction [2,3,4]. Plants employ various strategies to cope with Pi shortage, among which membrane phospholipid remodeling and phosphate recycling are key [5,6]. In this context, glycerophosphodiester phosphodiesterase (GDPD, also known as GPX-PDE) catalyzes the breakdown of glycerophosphodiesters into sn-glycerol-3-phosphate (G-3-P) and corresponding alcohols. GDPD genes have been identified in multiple species, including 13 in *Arabidopsis* [7], 13 in rice (*Oryza sativa* L.) [8], and 14 in maize (*Zea mays* L.) [9].

Beyond their established role in phosphorus homeostasis, *GDPD* genes have also been implicated in plant responses to abiotic stresses. In *Arabidopsis*, several *AtGDPD* genes are transcriptionally regulated by salt and osmotic stress [7], suggesting a broader function beyond phosphate deficiency. Abiotic stresses such as drought, salinity, and extreme temperatures severely impair crop growth and productivity. Plants counteract these adverse conditions through complex networks involving membrane lipid remodeling, signal transduction, and regulation of reactive oxygen species (ROS) [1,10]. Because GDPD enzymes hydrolyze glycerophosphodiesters derived from membrane phospholipids, they are strategically positioned to influence membrane integrity and potentially release signaling molecules under stress. However, the functional relevance of *GDPD* genes in cucumber under abiotic stress remains unknown. Moreover, recent evidence indicates that plant growth-promoting rhizobacteria (PGPR) can modulate host gene expression to enhance stress tolerance [11,12], but whether PGPR influence *GDPD* expression has never been investigated. Therefore, clarifying the expression patterns of cucumber *GDPD* genes under drought and salt stress, as well as their possible regulation by PGPR, is a critical step toward understanding their broader physiological roles.

Cucumber (*Cucumis sativus* L.) belongs to the Cucurbitaceae family and is an important economic crop that is widely cultivated around the world [13]. Although *GDPD* genes have been studied in various species, systematic investigation in cucumber is still lacking. In this study, we performed the first genome-wide identification of the *GDPD* gene family in cucumber, and comprehensively analyzed their phylogenetic relationships, gene structures, conserved motifs, synteny, promoter cis-elements, and selection pressure. We further investigated the expression patterns of *CsGDPD* genes under drought and salt stress, as well as their modulation by the plant growth promoting rhizobacterium GD17 (*Paraburkholderia* sp. GD17)—an aspect that has never been explored in any species. Through qRT-PCR validation, we identified *CsGDPD5* as a key stress-responsive gene dramatically induced by salt stress and partially attenuated by PGPR-GD17. These results provide evolutionary and functional insights into the cucumber *GDPD* family, and highlight a novel role of *GDPD* genes in PGPR-mediated abiotic stress tolerance.

## 2. Materials and Methods

### 2.1. Plant Culture and Treatment

Cucumber seeds (*Cucumis sativus* L. cv. Zhongnong 26) were obtained from the Institute of Vegetable and Flower Research, Chinese Academy of Agricultural Sciences. After sterilization with 75% ethanol, seeds were germinated in darkness at 25 °C for 48 h, then transplanted into seedling pots. Growth conditions: 26/18 °C (day/night), 14/10 h photoperiod, 12,000 lx light intensity, and a soil mixture of soil:vermiculite:perlite = 3:2:1.

Seven days after transplanting, uniform seedlings were divided into two groups: control (CT) and +PGPR. When the first true leaves emerged, a bacterial suspension of PGPR-GD17 (10^8^ cells/mL) was prepared by centrifuging a 48 h shaker culture and resuspending the pellet in pure water, then applied to the +PGPR group. A second identical inoculation was performed seven days later, followed by normal cultivation.

After the second inoculation, seedlings of similar size were assigned to six groups: CT, Drought, NaCl, +PGPR, PGPR + Drought, and PGPR + NaCl. Treatments began at the four-true-leaf stage. Drought stress was imposed by water withdrawal for 9 days. Salt stress was applied by irrigation with 100 mmol/L NaCl every 3 days for a total of two treatments. The third true leaf was sampled from each group for analysis.

### 2.2. Identification of Cucumber GDPD Genes

The HMM file (PF02704) of the *GDPD* gene family was downloaded from the InterPro database (https://www.ebi.ac.uk/interpro/ (accessed on 15 November 2025)) [14,15] and used to search for *GDPD* genes in the cucumber genome [16]. The protein sequences of the candidate *GDPD* genes were extracted from the Cucurbit Genomics Database (http://www.cucurbitgenomics.org/ (accessed on 15 November 2025)). All retrieved protein sequences were subsequently verified using the InterPro database and the NCBI database (https://www.ncbi.nlm.nih.gov/ (accessed on 15 November 2025)). Ultimately, six *GDPD* genes were identified in the cucumber genome [17].

### 2.3. Chromosomal Localization, Physicochemical Properties, and 3D Structure Prediction of GDPD Proteins

For chromosome distribution analysis, the GFF3 file of ChineseLong_V3 was downloaded from the Cucurbit Genomics Database, and the chromosomal localization map was generated using TBtools (v2.467) software [17]. For physicochemical characterization, the protein sequences of the six *CsGDPD* genes were submitted to the ProtParam online tool (https://web.expasy.org/protparam/ (accessed on 29 November 2025)) to determine protein length, instability index, and isoelectric point (pI) [18]. Subcellular localization was predicted using the CELLO v2.5 platform (http://cello.life.nctu.edu.tw/ (accessed on 1 December 2025)) [19]. For three-dimensional structure prediction, the protein sequences were submitted to the SWISS-MODEL server (https://swissmodel.expasy.org/ (accessed on 1 December 2025)), and models were generated based on the best matching templates [20]. Model quality was assessed using GMQE scores and Ramachandran plots.

### 2.4. Phylogenetic Tree of CsGDPD Genes

The evolutionary relationships among the *GDPD* gene family members in cucumber, *Arabidopsis*, and rice were analyzed through phylogenetic reconstruction. Using MEGA 12 software with the MUSCLE algorithm for multiple-sequence alignment, a maximum likelihood (ML) phylogenetic tree was generated under the best-fit substitution model (determined by ModelFinder) to represent their genetic divergence [21]. The resultant phylogenetic tree was subsequently visualized and optimized using the iTOL online platform (https://itol.embl.de/ (accessed on 5 December 2025)) [22].

### 2.5. Conserved Motif and Gene Structure Analysis

Conserved motifs of the cucumber GDPD proteins were identified using the MEME online tool (http://meme-suite.org/tools/meme (accessed on 12 December 2025)) with default parameters except for setting the maximum number of motifs to 10 and the motif length between 6 and 200 amino acids [23]. Gene structures were visualized based on the ChineseLong_V3 GFF3 file using the Gene Structure View tool in TBtools. All resulting visualizations were integrated and displayed using TBtools.

### 2.6. Synteny and Selection Pressure Analysis

Synteny analysis was performed to investigate collinear relationships of *GDPD* genes between cucumber and two reference species, *Arabidopsis* and rice. Using TBtools software, the genomic sequences and annotation files (GFF3) of cucumber (ChineseLong_V3), *Arabidopsis*, and rice were loaded. One-step synteny analysis was conducted with default parameters to identify collinear blocks containing *GDPD* genes. Syntenic gene pairs were visualized using the Dual Synteny Plotter tool in TBtools. To further evaluate the selective constraints acting on the orthologous gene pairs, selection pressure analysis was performed using MEGA11. The non-synonymous (dN) to synonymous (dS) substitution rate ratio (dN/dS) was calculated for each orthologous pair between cucumber and *Arabidopsis* GDPD genes. A dN/dS value less than 1, equal to 1, or greater than 1 indicates purifying selection, neutral evolution, or positive selection, respectively.

### 2.7. Cis-Acting Elements Analysis of Cucumber GDPD Genes

The 2.0 kb upstream promoter sequences of the cucumber *GDPD* genes were extracted from the transcription start site using TBtools software. Cis-acting elements in the promoter regions were identified using the PlantCare online database (https://bioinformatics.psb.ugent.be/webtools/plantcare/html/ (accessed on 8 January 2025)) [24]. The results were visualized and displayed using TBtools.

### 2.8. Tissue-Specific Expression Analysis

The transcriptome data of cucumber tissues (PRJNA80169) were obtained from the Cucurbit Genomics Database and combined with the cucumber ChineseLong_V3 genome for RNA-seq reanalysis. The expression heatmap of *CsGDPD* genes across tissues was visualized using ChiPlot (https://www.chiplot.online/ (accessed on 28 January 2025)).

### 2.9. RNA Isolation, cDNA Synthesis, and Quantitative Real-Time PCR Analysis

Total RNA was extracted from cucumber samples using a universal plant total RNA extraction kit (Promega, Madison, WI, USA). First-strand cDNA was synthesized from 1 μg of total RNA using the PrimeScript RT reagent kit (TaKaRa, Kusatsu, Japan) according to the manufacturer’s instructions. Quantitative real-time PCR (qRT-PCR) was performed on a LightCycler 96 system (Roche, Basel, Switzerland) using TB Green Premix Ex Taq (Takara). The reaction mixture (20 μL) contained 10 μL of 2× TB Green Premix, 0.4 μM of each gene-specific primer, and 2 μL of diluted cDNA (equivalent to 50 ng of input RNA). The thermal cycling conditions were: 95 °C for 30 s, followed by 40 cycles of 95 °C for 5 s and 60 °C for 30 s. Melting curve analysis was conducted to verify amplification specificity, and primer amplification efficiency was determined by standard curve analysis using a dilution series of cDNA. The cucumber *Actin* gene (*CsActin*) was used as an internal reference. Relative expression levels of each target gene were calculated using the 2^−ΔΔCT^ method. The expression level of the control group (CT) was set to 1, and the expression levels of all treatment groups were normalized relative to CT. Three biological replicates were performed for each treatment, and each reaction was run in technical triplicates. The sequences of gene-specific primers are listed in Supplementary Table S2.

## 3. Results

### 3.1. Identification and Characterization of GDPD Genes

**Figure 1:**
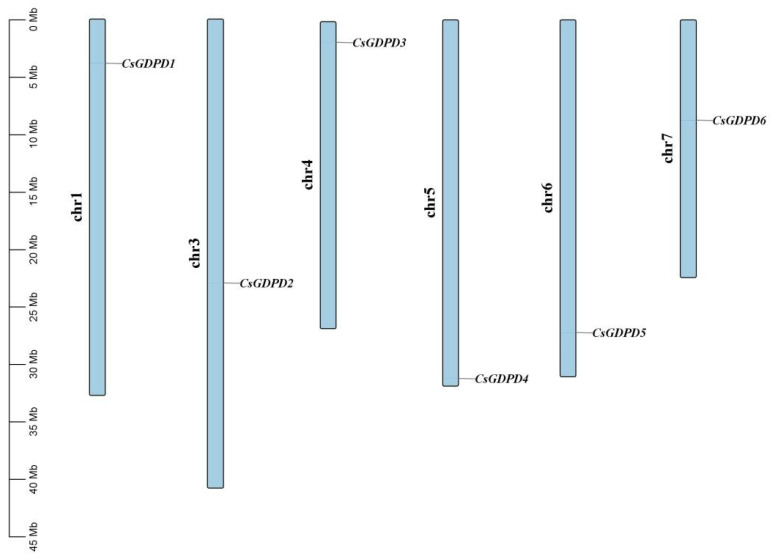
The distribution and localization of *CsGDPDs* on chromosomes are shown, with chromosome names and the scale in megabases (Mb) displayed to the left of each chromosome.

A total of six *GDPD* genes were identified in the cucumber genome (ChineseLong_V3). These genes, named *CsGDPD1* to *CsGDPD6*, were located on chromosomes 1, 3, 4, 5, 6, and 7, respectively (Figure 1).

The protein length ranged from 330 (CsGDPD4) to 764 (CsGDPD6) amino acids, and the molecular weight ranged from 37.30 kDa (CsGDPD4) to 83.50 kDa (CsGDPD6). The theoretical isoelectric points (pI) ranged from 4.99 (CsGDPD2) to 9.74 (CsGDPD4). Instability index analysis indicated that CsGDPD1 and CsGDPD5 were unstable (instability index > 40), whereas the other four GDPD proteins were stable. The grand average hydropathicity (GRAVY) values of CsGDPD1, CsGDPD2, CsGDPD3, and CsGDPD4 were less than zero, indicating that they are hydrophilic, while the remaining two were hydrophobic. Subcellular localization prediction showed that two *GDPD* genes were located in the cytoplasm, two in the plasma membrane, and the remaining two in the extracellular space and mitochondria, respectively (Table 1).

To gain insight into the structural conservation of the CsGDPD proteins, their three-dimensional structures were predicted using SWISS-MODEL (Figure S1). For all six proteins, reliable models were obtained with template sequence identity ranging from 73.3% to 100% and GMQE scores between 0.82 and 0.89, indicating high modeling quality. Ramachandran plot analysis showed that over 92% of the residues were in favored regions, further validating the structural reliability. The predicted structures of all six CsGDPD proteins were spatially similar, suggesting conserved biochemical functions.

### 3.2. Gene Structure and Analysis of Conserved Motifs

Based on the conserved motif composition of the six cucumber *GDPD* proteins, they were divided into two groups (Group I and Group II). Group I included *CsGDPD1*, *CsGDPD4*, *CsGDPD5*, and *CsGDPD6*, while Group II included *CsGDPD2* and *CsGDPD3*.

**Figure 2:**
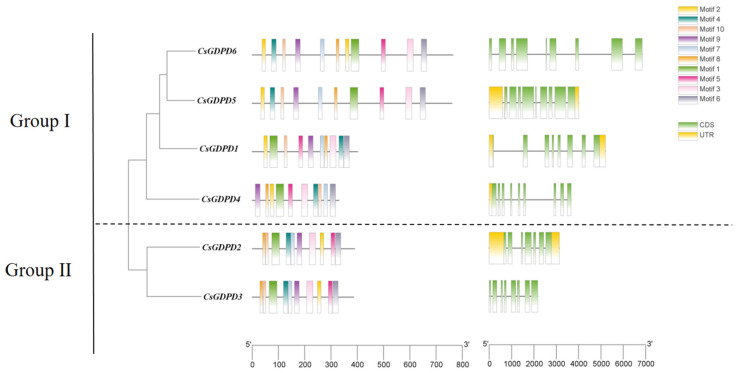
Schematic diagram of the exon–intron structures of *GDPD* genes and conserved motifs of GDPD proteins in cucumber.

Motif analysis using MEME revealed that *CsGDPD6* contained 11 conserved motifs, whereas the other five members each contained 10 motifs (Figure 2). In Group II, the two genes shared identical motif types and order, suggesting they may perform similar biological functions. In contrast, Group I members exhibited variable motif patterns, and their motif composition differed significantly from that of Group II, which may account for the functional diversification within this family. In addition, gene structure analysis showed that the number of exons ranged from 7 to 9, and the number of introns ranged from 6 to 8. Specifically, *CsGDPD4* and *CsGDPD6* both contained 9 exons and 8 introns (Figure 2).

### 3.3. Phylogenetic Analysis

**Figure 3:**
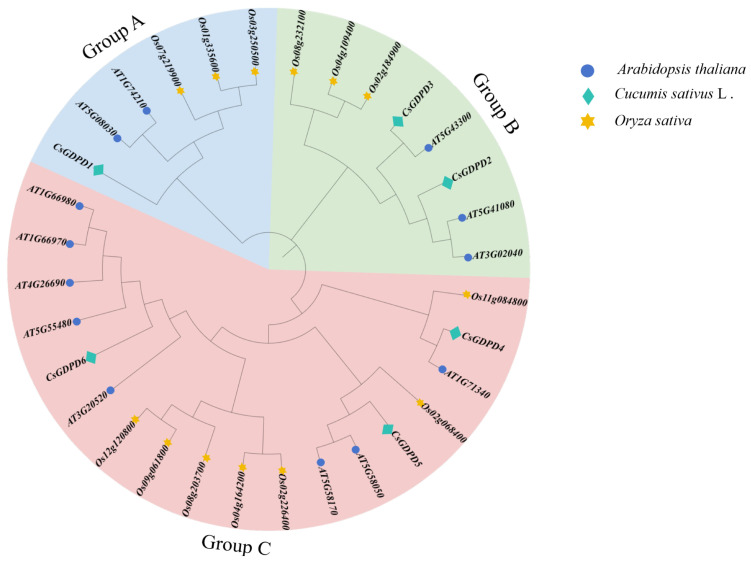
Phylogenetic tree of GDPD proteins from cucumber, *Arabidopsis*, and rice. Green diamonds, blue circles, and yellow five-pointed stars represent GDPD members from cucumber, *Arabidopsis*, and rice, respectively.

A phylogenetic tree was constructed using the GDPD protein sequences of cucumber (6), *Arabidopsis* (13), and rice (13) (Figure 3). The tree was divided into three groups (Groups A, B, and C), containing 6, 8, and 18 *GDPD* genes, respectively. Two pairs of putative orthologous genes were identified between cucumber and *Arabidopsis*, *CsGDPD3* with *AT5G43300*, and *CsGDPD4* with *AT1G71340*. No direct orthologous relationship between cucumber and rice *GDPDs* was observed in the phylogenetic tree. *CsGDPD1* clusters tightly with *AT1G74210* and *AT5G08030* in Group A, whereas *CsGDPD2* is placed into Group B, clustering closely with *AT3G02040* and *AT5G41080*. This suggests that *CsGDPD1* and *CsGDPD2* may have retained conserved functions. Although *CsGDPD5* and *CsGDPD6* both belonged to Group C, they clustered with different *Arabidopsis* genes, indicating possible species-specific functional divergence in cucumber. Based on the close evolutionary relationships, the potential biological functions of cucumber *GDPD* genes may be inferred from their well-characterized counterparts in *Arabidopsis*.

### 3.4. Synteny Analysis of GDPD Genes Among Arabidopsis, Rice and Cucumber and Selection Pressure Analysis

**Figure 4:**
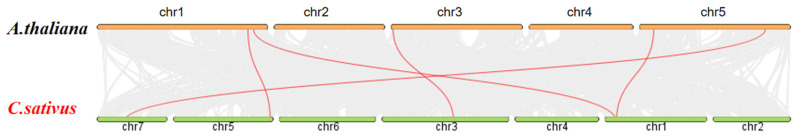
Chromosomal collinearity relationships between Cucumber and *Arabidopsis.* Gray lines in the background indicate collinear blocks in Cucumber and *Arabidopsis* genomes, while the red lines represent the *CsGDPD*.

To further investigate the evolutionary conservation of the *CsGDPD* gene family, synteny analysis was performed between cucumber and two reference species, *Arabidopsis* and rice (Figure 4). In total, five syntenic *GDPD* gene pairs were identified between cucumber and *Arabidopsis*, *CsGDPD1* exhibited synteny with *AT1G74210* and *AT5G08030*; *CsGDPD2* with *AT3G02040*; *CsGDPD4* with *AT1G71340*; and *CsGDPD6* with *AT5G55480*. No syntenic relationships were detected between cucumber and rice *GDPD* genes. The synteny results are largely consistent with the phylogenetic relationships described. The absence of syntenic counterparts for *CsGDPD3* and *CsGDPD5* in both *Arabidopsis* and rice may reflect species-specific evolution or rapid divergence, consistent with their distinct phylogenetic positions (e.g., *CsGDPD3* as a putative ortholog of *AT5G43300* but without collinearity). Overall, the combination of phylogenetic and syntenic analyses provides complementary evidence for the evolutionary trajectories of the cucumber *GDPD* gene family.

To further assess the evolutionary conservation between cucumber and *Arabidopsis GDPD* genes, the dN/dS ratio was calculated for orthologous pairs (Table S1). All dN/dS values were below 1, indicating that these genes have evolved under purifying selection and have retained similar functions across species. Notably, the low dN/dS values for *CsGDPD1* with its *Arabidopsis* counterparts (*AT1G74210* and *AT5G08030*) suggest that these genes have been highly conserved during evolution and may share essential, irreplaceable roles. Similarly, *CsGDPD4* and its ortholog *AT1G71340* also showed strong purifying selection, supporting their functional similarity. In contrast, *CsGDPD3* and *CsGDPD4* with *AT5G43300* exhibited relatively higher dN/dS values, implying slightly relaxed but still conserved functions. Overall, the dN/dS analysis reinforces the conclusion from the phylogenetic tree that cucumber *GDPD* genes and their *Arabidopsis* homologs are likely to perform similar biological functions, providing a molecular basis for functional prediction in cucumber.

### 3.5. Analysis of the Cis-Acting Elements in Cucumber GDPD Genes

**Figure 5:**
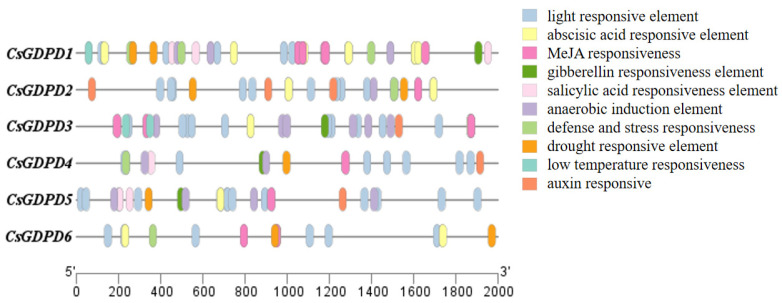
The cis-acting elements analysis of the promoters of cucumber *GDPD* genes.

To predict the potential regulatory mechanisms of the *CsGDPD* genes, the 2000 bp upstream promoter sequences of all six *CsGDPD* genes were analyzed using PlantCare (Figure 5). A total of 10 types of cis-acting elements were identified, including light-responsive elements, MeJA-responsive elements, anaerobic induction elements, hormone-responsive elements (auxin, abscisic acid, MeJA, and gibberellin), and stress-responsive elements (low temperature, drought, defense, and salt). All six genes contained light-responsive, MeJA-responsive, and anaerobic induction elements. The number and distribution of cis-elements varied among family members. *CsGDPD1* contained the highest number of elements, while *CsGDPD2* contained the fewest. Genes such as *CsGDPD1*, *CsGDPD3*, and *CsGDPD5* exhibited a higher density of stress and hormone-related elements, suggesting their potential involvement in multiple signaling pathways. Notably, drought-responsive elements are present in all *CsGDPD*, leading us to speculate that *CsGDPDs* may play important roles in plant stress resistance. Overall, the promoter analysis indicates that the *CsGDPD* gene family may participate in various biological processes, including hormone signal transduction, stress adaptation, and light response.

### 3.6. Tissue-Specific Expression Analysis of GDPD Genes

**Figure 6:**
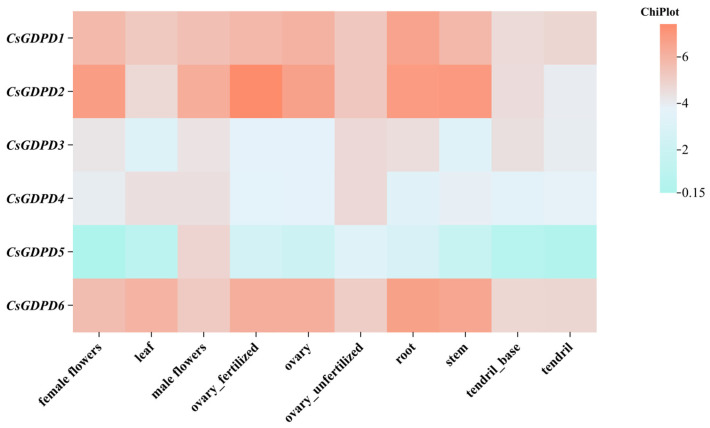
The expression profiling of cucumber *GDPD* genes in different tissues.

The expression profiles of the six *CsGDPD* genes across ten different cucumber tissues were analyzed using publicly available transcriptome data (Figure 6). *CsGDPD1*, *CsGDPD2*, and *CsGDPD6* showed generally high expression levels in all tissues, whereas *CsGDPD3*, *CsGDPD4*, and *CsGDPD5* exhibited low expression across most tissues. Notably, *CsGDPD5* was predominantly expressed in male tissues, and *CsGDPD4* showed relatively higher expression in leaf, male, and unfertilized ovary tissues. *CsGDPD2* was highly expressed in all tissues except tendril. Interestingly, the expression levels of *CsGDPD1* and *CsGDPD2* were significantly higher in female flowers than in male flowers, suggesting a potential role in female reproductive organ development and fruit setting. The presence of hormone-responsive elements in the promoters of these genes (Figure 5) may partly explain their differential expression in floral tissues. These tissue-specific patterns provide clues for further functional studies of the *CsGDPD* family.

### 3.7. qRT-PCR Expression Analysis of CsGDPD Genes Under Drought, Salt, and PGPR-GD17 Treatments

**Figure 7:**
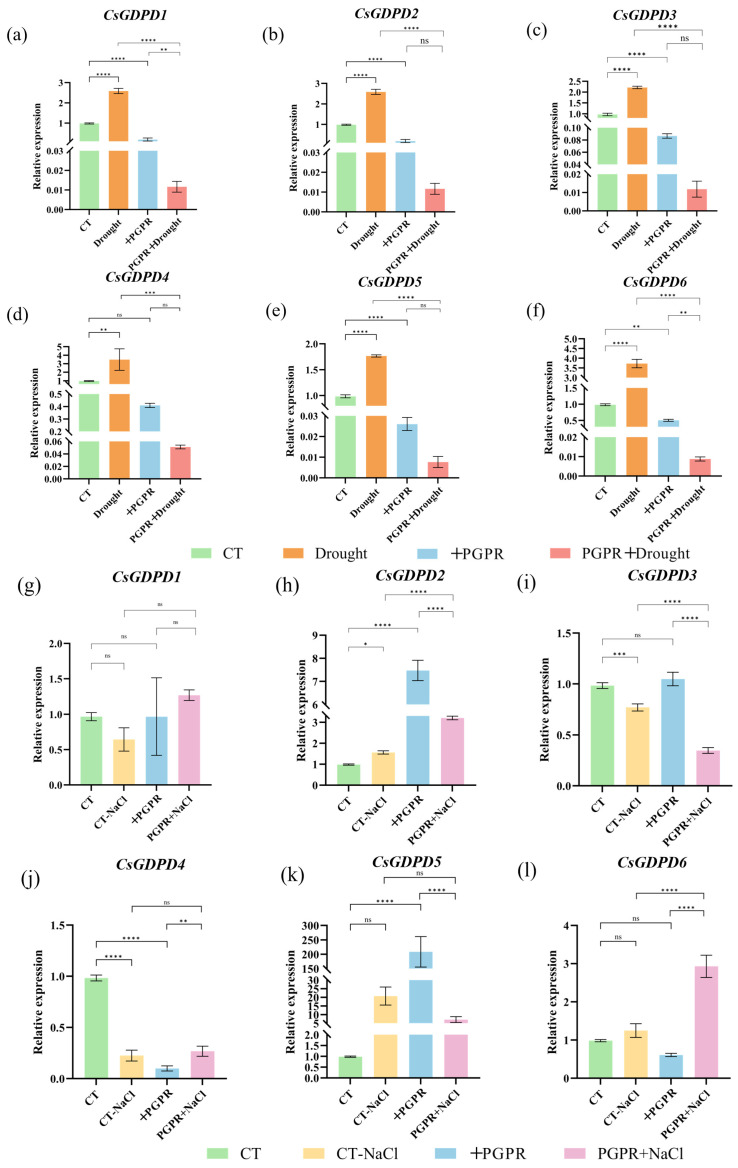
Expression profiles of *CsGDPD* genes in response to various stress. (**a**–**f**) Relative expression levels of six *CsGDPD* genes in cucumber leaves under control (CT), drought (Drought), +PGPR, and combined drought + GD17 (PGPR + Drought) conditions. (**g**–**l**) Relative expression levels of six *CsGDPD* genes under control (CT), salt stress (NaCl), and combined salt + GD17 (PGPR + NaCl) conditions. Data are presented as mean ± SE of three biological replicates. Significant differences from CT are indicated by asterisks (one-way ANOVA, “ns” indicates insignificant, * *p* < 0.05, ** *p* < 0.01, *** *p* < 0.001, **** *p* < 0.0001,).

To investigate the effects of the PGPR-GD17 on *CsGDPD* expression under abiotic stresses, qRT-PCR was performed under drought and salt stress, with or without PGRP-GD17 inoculation (Figure 7). Under drought stress alone (D), all six *CsGDPD* genes were upregulated compared with the control (CT), with expression levels ranging from 1.77 (*CsGDPD5*) to 3.73 (*CsGDPD6*), indicating a general positive response of the family to drought. Inoculation with +PGPR strongly suppressed the expression of all six genes, with values ranging from 0.026 (*CsGDPD5*) to 0.50 (*CsGDPD6*), suggesting that PGPR-GD17 negatively regulates *CsGDPD* transcription under normal conditions. In the PGPR + Drought group, the expression levels of all genes were further reduced to extremely low levels (0.007–0.051), even lower than with +PGPR, indicating a synergistic suppressive effect between PGPR-GD17 and drought. Under salt stress alone (+NaCl), the responses were gene-specific, *CsGDPD1*, *CsGDPD3*, *CsGDPD4*, and *CsGDPD6* showed moderate changes (0.22–1.56), while *CsGDPD5* was dramatically upregulated (20.39), and *CsGDPD2* also increased (1.56). PGPR-GD17 under salt conditions (PGPR + NaCl) partially reversed the salt-induced changes, *CsGDPD1*, *CsGDPD2*, *CsGDPD3*, and *CsGDPD6* were further induced (1.27–3.20), whereas *CsGDPD4* and *CsGDPD5* were reduced compared with salt alone but remained higher than control for *CsGDPD5* (7.06). The salt-responsive expression of *CsGDPD5* and its altered expression under PGPR-GD17 treatment identify it as a candidate for stress tolerance, but further functional studies (e.g., gene editing or overexpression) are necessary to establish its actual role.

## 4. Discussion

In this study, we performed the first genome-wide identification and systematic characterization of the *GDPD* gene family in cucumber. A total of six *GDPD* genes were identified in the cucumber genome, which is fewer than in *Arabidopsis* (13), rice (13) and maize (14) [7,8,9]. The smaller size of the cucumber *GDPD* family may reflect species-specific gene loss or differential expansion during evolution, as has been observed for other gene families in Cucurbitaceae [25,26].

Phylogenetic analysis showed that the six *CsGDPD* genes were distributed among three groups (A, B and C). Consistent with the evolutionary distance between dicots and monocots, cucumber GDPD proteins clustered more closely with those of *Arabidopsis* (dicot) than with those of rice (monocot). Two pairs of putative orthologs were identified between cucumber and *Arabidopsis* (*CsGDPD3*/*AT5G43300* and *CsGDPD4*/*AT1G71340*). Synteny analysis further revealed five collinear gene pairs between cucumber and *Arabidopsis*, but no syntenic relationship with rice. These results indicate that the cucumber GDPD family shares a closer evolutionary history with the model dicot. The absence of syntenic relationships between cucumber and rice *GDPD* genes is consistent with the deep evolutionary divergence between dicots and monocots, which are estimated to have separated approximately 140–150 million years ago [27]. During this long period, extensive genome rearrangements, including chromosomal fusions, inversions, translocations, and differential gene loss, may have disrupted ancestral collinearity [28]. Lineage-specific expansion or contraction of gene families could also have led to the loss of syntenic signals while maintaining functional orthology at the sequence level [29]. Similar phenomena have been observed for other gene families in Cucurbitaceae when compared with monocot genomes [30]. The absence of syntenic counterparts for *CsGDPD3* and *CsGDPD5* in both *Arabidopsis* and rice suggests that these two genes might have originated from species-specific duplication or rapid divergence, potentially leading to neo-functionalization [31].

Selection pressure analysis (dN/dS) revealed that all orthologous pairs between cucumber and *Arabidopsis* have dN/dS values below 1, indicating strong purifying selection [32,33]. This supports the notion that the GDPD family has maintained conserved enzymatic functions during evolution. Notably, *CsGDPD1*, with its *Arabidopsis* counterparts (*AT1G74210* and *AT5G08030*), exhibited extremely low dN/dS values, implying essential, non-redundant roles. The higher dN/dS values for *CsGDPD3* and *CsGDPD4* with *AT5G43300* suggest slightly relaxed constraints, which may allow for functional divergence [7]. Beyond evolutionary conservation, our expression data provide insights into how CsGDPD proteins may contribute to drought and salt tolerance at the physiological and molecular levels. Under drought stress, all six *CsGDPD* genes were upregulated (1.5- to 3.5-fold), and under salt stress, *CsGDPD5* was dramatically induced (20-fold), as shown by qRT-PCR. These expression patterns are consistent with the presence of drought-responsive elements (DRE) and abscisic acid-responsive elements (ABRE) in their promoters (Figure 5). The GDPD enzyme hydrolyzes membrane-derived glycerophosphodiesters to produce sn-glycerol-3-phosphate (G-3-P) and free alcohols such as choline. G-3-P can serve as a precursor for the synthesis of glycerol, a compatible osmolyte that stabilizes membranes and proteins under osmotic stress [34]. Additionally, choline released from glycerophosphocholine can be converted into glycine betaine, a well-known osmoprotectant that maintains cellular water balance and protects macromolecular structures during drought and salinity [35]. Therefore, the stress-induced upregulation of *CsGDPD* genes may enhance the production of these osmoprotectants, thereby contributing to cellular adaptation. Furthermore, G-3-P has been implicated in stress signaling pathways, potentially linking GDPD activity to broader regulatory networks. While direct biochemical evidence is still lacking in cucumber, our transcriptional and promoter analyses strongly suggest that CsGDPD proteins participate in abiotic stress tolerance through the generation of osmoprotective metabolites and maintenance of membrane integrity.

The promoter cis-acting element analysis revealed that all *CsGDPD* genes contain multiple stress-responsive elements, including drought-responsive elements (DRE) and abscisic acid-responsive elements (ABRE), as well as hormone-responsive elements (MeJA, auxin, gibberellin). This is consistent with our qRT-PCR results: under drought stress, all six *CsGDPD* genes were significantly upregulated (1.5- to 3.5-fold), and under salt stress, *CsGDPD5* was dramatically induced (20-fold). Notably, *CsGDPD1*, *CsGDPD3*, and *CsGDPD5* exhibited a higher density of stress- and hormone-related elements in their promoters, which correlates with their relatively higher expression levels under stress conditions. For instance, *CsGDPD5*, the most strongly salt induced gene, possesses multiple ABRE and DRE motifs, suggesting that these elements may directly contribute to its transcriptional activation. These observations are in line with previous reports that DRE and ABRE are key regulators of stress-inducible gene expression [36,37]. Collectively, our promoter and expression data strongly indicate that the *CsGDPD* genes are transcriptionally regulated by drought and salt stress, likely through the action of these cis-elements. The presence of light-responsive and anaerobic induction elements further suggests additional roles in photosynthesis and hypoxia adaptation, which warrants future investigation.

Tissue-specific expression analysis revealed that *CsGDPD1*, *CsGDPD2* and *CsGDPD6* are broadly expressed, whereas *CsGDPD4* and *CsGDPD5* show preferential expression in reproductive tissues (male flowers, unfertilized ovaries). This pattern is similar to that observed for *CaGDPD1* in pepper and several *AtGDPD* genes in *Arabidopsis*, which are highly expressed in floral organs and are implicated in reproductive development [7,8]. The elevated expression of *CsGDPD1* and *CsGDPD2* in female flowers compared with male flowers suggests a potential role in fruit setting and female reproductive organ development, which deserves further functional investigation. However, whether cucumber CsGDPD4 and CsGDPD5 actually play similar roles remains unknown and requires experimental validation.

PGPR-GD17 is a PGPR that has been experimentally validated to play an important regulatory role in drought tolerance of cucumber [38]. Therefore, we investigated the effect of PGPR-GD17 on the expression of *CsGDPD* genes under drought and salt stress. qRT-PCR under drought and salt stress, with or without the PGPR-GD17, provided novel insights into the regulation of *CsGDPD* genes. Drought alone upregulated all six *CsGDPD* genes, indicating a positive response to dehydration. This is in line with the presence of drought-responsive elements in their promoters. Interestingly, +PGPR strongly suppressed *CsGDPD* expression under normal conditions, and combined PGPR + Drought led to synergistic suppression at extremely low levels. In contrast, under salt stress, PGPR-GD17 partially reversed salt-induced changes: *CsGDPD5* was dramatically induced (20-fold) by salt alone, and PGPR-GD17 reduced this induction but still maintained higher expression than the control. These differential responses suggest a possible regulatory effect of PGPR-GD17. The strong and specific upregulation of *CsGDPD5* under salt stress highlights it as a prime candidate for salt tolerance breeding. The suppressive effect of PGPR-GD17 under drought might reflect a trade-off between growth promotion and stress signaling, or it could indicate that PGPR-GD17 helps the plant avoid over-activation of phospholipid remodeling pathways under combined stress [5].

Overall, this study provides a foundation for understanding the evolution and regulation of the cucumber GDPD family. The salt-responsive expression of *CsGDPD5* and its correlation with PGPR-GD17 treatment suggest a possible role for this gene in stress tolerance; however, direct functional validation (e.g., via gene editing or overexpression) is required to confirm its involvement.

## 5. Conclusions

In this study, six GDPD family genes were systematically identified in the cucumber genome. They are distributed on chromosomes 1, 3, 4, 5, 6, and 7, and encode proteins with conserved three-dimensional structures. Phylogenetic and dN/dS selection pressure analyses demonstrated that cucumber *GDPD* genes are evolutionarily close to their *Arabidopsis* homologs and have evolved under purifying selection, supporting functional conservation. Synteny analysis identified five collinear gene pairs between cucumber and *Arabidopsis*, but no synteny with rice. Promoter cis-acting element analysis revealed abundant stress and hormone-responsive elements, suggesting involvement in multiple signaling pathways. Tissue-specific expression profiling showed that *CsGDPD1*, *CsGDPD2*, and *CsGDPD6* are broadly expressed, while *CsGDPD4* and *CsGDPD5* exhibit preferential expression in reproductive tissues, implying specialized roles in growth and development. qRT-PCR under drought and salt stress, with or without PGPR-GD17, uncovered that drought alone upregulates all *CsGDPD* genes; +PGPR suppresses them; and combined GD17 + Drought causes synergistic suppression. Under salt stress, *CsGDPD5* is massively induced (20-fold), and PGPR-GD17 partially modulates this response. These results suggest that CsGDPD5 could be a candidate for further investigation in salt tolerance breeding, but this remains to be validated by functional studies. The data also indicate that PGPR-GD17 may exert gene-specific and stress-dependent regulation. Overall, this study provides essential gene resources and a theoretical basis for further functional analysis of *GDPD* genes in cucumber resistance breeding.

## Figures and Tables

**Table 1:** List of six *CsGDPD* genes and basic characterizations.

Gene ID (v3)	Gene ID (v4)	Gene Name	Location	Protein Length (aa)	MW (Da)	PI	Instability Index	GRAVY Value	SC Localization
*CsaV3_1G006010*	*CsaV4_1G000648*	*CsGDPD1*	Chr 1	402	46,177.46	5.72	35.53	−0.272	Extracellular
*CsaV3_3G026730*	*CsaV4_3G002610*	*CsGDPD2*	Chr 3	390	43,958.37	4.99	40.71	−0.206	Cytoplasmic
*CsaV3_4G002900*	*CsaV4_4G000298*	*CsGDPD3*	Chr 4	386	43,380.86	5.13	51.01	−0.165	Cytoplasmic
*CsaV3_5G039470*	*CsaV4_5G003422*	*CsGDPD4*	Chr 5	330	37,304.19	9.74	44.27	−0.202	Mitochondrial
*CsaV3_6G046000*	*CsaV4_6G003441*	*CsGDPD5*	Chr 6	760	83,290.67	5.23	37.57	0.067	Plasma Membrane
*CsaV3_7G018770*	*CsaV4_7G001093*	*CsGDPD6*	Chr 7	764	83,495.99	5.10	43.91	0.027	Plasma Membrane

Note: The databases used in the experiment were all Chinese long_v3 gene databases, and “Chr” is the abbreviation of “Chromosome”.

## Data Availability

The original data presented in the study are openly available in Cucurbit Genomics Database [accession number PRJNA80169].

## Supplementary Materials

The following supporting information can be downloaded at https://www.mdpi.com/article/10.3390/cimb48060602/s1.
