# Supplementary figures and images for: Genome-Wide Identification and Expression Analysis of the GDPD Gene Family in Cucumber (Cucumis sativus L.)
Source: Curr Issues Mol Biol. 2026 Jun 5;48(6):602. doi: 10.3390/cimb48060602 (PMC13298316; doi:10.3390/cimb48060602)

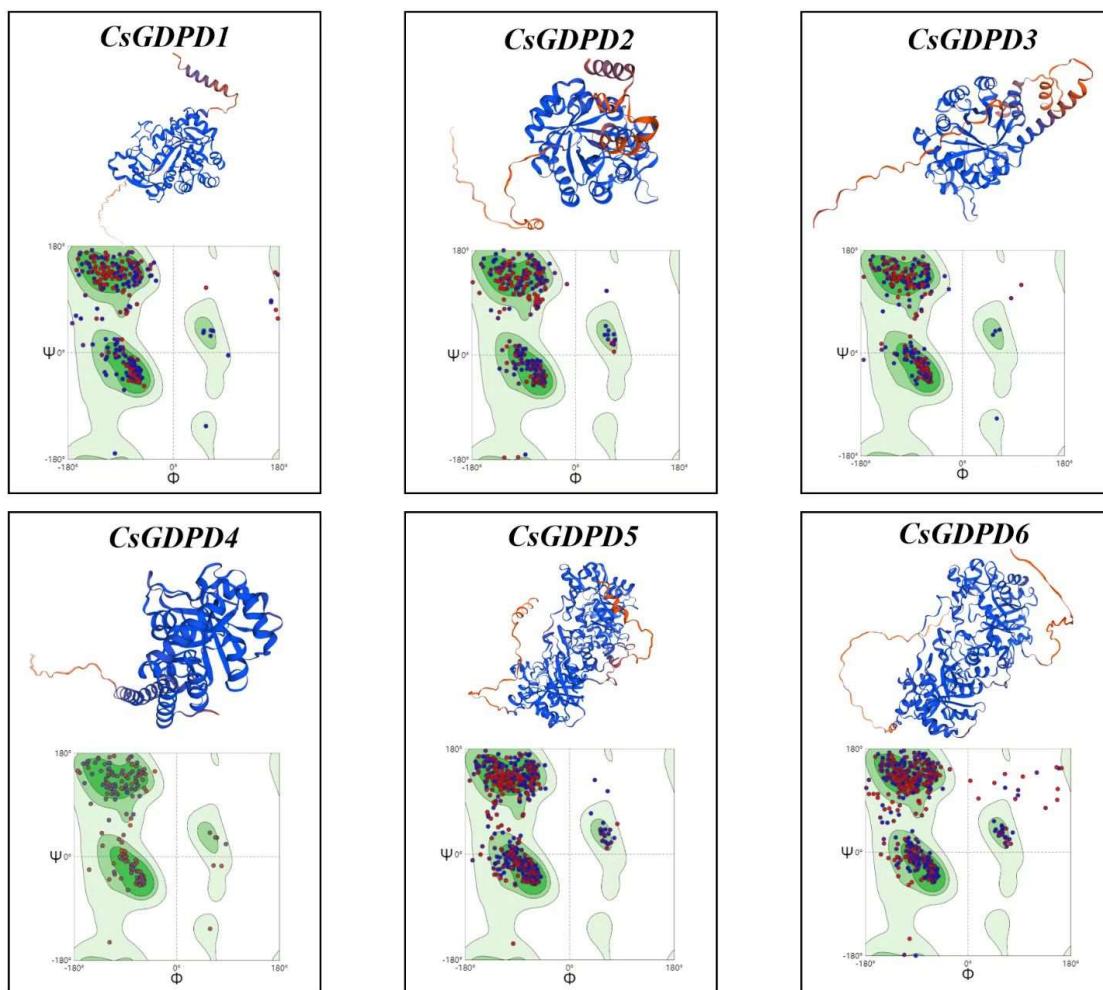

Figure S1: Predicted three-dimensional structures of the six CsGDPD proteins.

Supplement: Supplementary file 1 [file cimb-48-00602-s001.zip › Figure S1.pdf]
